# Navigating multimodal machine learning in papillary thyroid carcinoma: perspectives on pathomics and radiomics integration in thyroidology

**DOI:** 10.1016/j.clinsp.2026.101120

**Published:** 2026-10-01

**Authors:** Ilker Sengul, Demet Sengul

**Affiliations:** a Thyroidology Unit, Giresun University Faculty of Medicine, Turkey; b Division of Endocrine Surgery, Giresun University Faculty of Medicine, Turkey; c Department of General Surgery, Giresun University Faculty of Medicine, Turkey; d Department of Pathology, Giresun University Faculty of Medicine, Turkey

Dear Editor,

The rising global incidence of thyroid malignancies, particularly papillary thyroid carcinoma (PTC), necessitates advancements in diagnostic precision to mitigate the inherent subjectivity of traditional risk stratification systems such as C-TIRADS, and TBSRTC in thyroidology. In this context, the study by Pang et al.^1^ provides a rigorous methodological framework for “intelligent pathological diagnosis” by synthesizing high-resolution digital pathomics with ultrasound radiomics. By leveraging 40 × magnification digital cytopathology and meticulous 3D Slicer-based lesion delineation, they have captured the nuanced morphological and textural signatures ‒ such as nuclear architecture and voxel distribution ‒ that frequently elude conventional visual inspection.

While the reported area under the curve (AUC) of 0.9907 for the support vector machine (SVM) model is technically impressive, several methodological considerations warrant further discussion for clinical translation. Antecedently, the extraction of 37 optimal features from a sample size of 385 cases raises questions regarding potential overfitting. It would be of significant scholarly interest to determine if the authors performed feature stability assessments, such as bootstrapping, to ensure the robustness of these signatures across different cross-validation folds.

Furthermore, the study utilized cytological slides from fine-needle aspiration (FNA), yet the inherent variability in specimen quality (e.g., cell density and staining artifacts) remains a clinical bottleneck. While the model shows preliminary promise, its reliance on a retrospective, single-center dataset limits its current generalizability. To catalyze a definitive paradigm shift in thyroid oncology, it is imperative that future research move beyond a preliminary model through prospective validation across diverse imaging platforms and multi-institutional cohorts to ensure the generalizability and algorithmic resilience of this multi-omics integration.

In conclusion, Pang et al.^1^ offer a valuable exploration of multi-omics integration in thyroidology.^2^, ^3^ Addressing feature resilience and conducting external validation will be critical steps in evolving these algorithms from technical prototypes into reliable clinical decision-support tools for thyroid oncology.

## Data availability statement

Data sharing is not applicable to this correspondence as no new datasets were generated or analyzed during the current work; however, inquiries regarding the scientific discourse may be directed to the corresponding author.

## Ethics approval

Not applicable.

## Authors’ contributions

Both authors contributed equally to the conceptualization, writing, and revision of this correspondence.

## Funding

None declared.

## Conflicts of interest

The authors declare no conflicts of interest.
